# Clinical significance of circulating soluble immune checkpoint proteins in sorafenib-treated patients with advanced hepatocellular carcinoma

**DOI:** 10.1038/s41598-020-60440-5

**Published:** 2020-02-25

**Authors:** Minh Phuong Dong, Masaru Enomoto, Le Thi Thanh Thuy, Hoang Hai, Vu Ngoc Hieu, Dinh Viet Hoang, Ayako Iida-Ueno, Naoshi Odagiri, Yuga Amano-Teranishi, Atsushi Hagihara, Hideki Fujii, Sawako Uchida-Kobayashi, Akihiro Tamori, Norifumi Kawada

**Affiliations:** 1 Department of Hepatology, Graduate School of Medicine, Osaka City University, Osaka, Japan; 2 Department of Hepatology, Osaka City General Hospital, Osaka, Japan

**Keywords:** Tumour immunology, Hepatocellular carcinoma, Predictive markers

## Abstract

In hepatocellular carcinoma (HCC), the clinical significance of soluble immune checkpoint protein levels as predictors of patient outcomes or therapeutic responses has yet to be defined. This study profiled the baseline levels of sixteen soluble checkpoint proteins and their changes following sorafenib treatment for HCC. Plasma samples were obtained from 53 patients with advanced HCC at baseline, week 1, 2 and 4 of sorafenib treatment and tested the concentrations of 16 soluble checkpoint proteins using multiplexed fluorescent bead-based immunoassays. Multivariate analysis showed high sBTLA levels at baseline were an independent predictor of poor overall survival (p = 0.038). BTLA was highly expressed in T cells and macrophages in peritumoral areas. At week 2, sCD27 levels were decreased compared to baseline. By contrast, the concentrations of most inhibitory proteins, including sBTLA, sLAG-3, sCTLA-4, sPD-1, sCD80, sCD86 and sPD-L1, had significantly increased. The fold-changes of soluble checkpoint receptors and their ligands, including sCTLA-4 with sCD80/sCD86, sPD-1 with sPD-L1; and the fold-changes of sCTLA-4 with sBTLA or sPD-1 were positively correlated. sBTLA may be a good biomarker for predicting overall survival in HCC patients. Sorafenib treatment in patients with advanced HCC revealed dynamic changes of soluble checkpoint protein levels.

## Introduction

Immune checkpoint inhibitors, including monoclonal antibodies that target inhibitory immune receptors such as programmed cell death-1 (PD-1), programmed death ligand-1 (PD-L1) and cytotoxic T-lymphocyte-associated antigen-4 (CTLA-4), have emerged as a promising treatments for many types of cancer^1^. However, the preparation of antibodies is very costly, and therapeutic responses to antibody treatment only occur in a minority of patients. Further, reliable biomarkers that identify patients who would benefit from immunotherapy have yet to be identified. Several biomarkers, including high tumor mutational burden, PD-L1 and/or PD-1 expression, and the presence of tumor-infiltrating lymphocytes in the tumor microenvironment, have been extensively studied as predictors of responses to therapy^2^. In addition, circulating soluble immune checkpoint proteins, which are part of a family of full-length receptors produced by mRNA expression or by the cleavage of membrane-bound proteins, have been evaluated as non-invasively derived markers in various cancers, but not in hepatocellular carcinoma (HCC)^3^.

Primary liver cancer, predominantly HCC, is the second most common cause of cancer deaths globally^4^. Patients with early-stage HCC can receive potentially curative treatments, such as surgical resection, transplantation or ablation, and patients at the intermediate stage can receive chemoembolization. However, systemic therapies are normally indicated for those at the advanced stage^5,6^. Two molecular-targeted therapies, the anti-angiogenic kinase inhibitors sorafenib^7^ and lenvatinib^8^, are recommended as first-line treatments for patients with advanced HCC who have well-preserved liver function. Other anti-angiogenic agents, including regorafenib^9^, cabozantinib^10^ and ramucirumab (if α-fetoprotein >400 ng/mL)^11^, have been approved as second-line treatments. In addition, immune checkpoint inhibitors, such as nivolumab^12^ and pembrolizumab^13^, may be used for patients who were refractory to prior sorafenib therapy.

Clinical trials that combine the use of an anti-angiogenic agent with an immune checkpoint inhibitor are ongoing; for example, a combination of lenvatinib and pembrolizumab is currently being tested in multiple cancer types^14^. In HCC, combinations of bevacizumab plus atezolizumab^15^ and apatinib plus camrelizumab^16^ are also under clinical investigations. The design and rationale of these combination therapy studies are based on results of previous *in vivo* studies, indicating that anti-angiogenic agents may enhance anti-tumor immunity through multiple mechanisms, such as increase in dendritic cell maturation, T cell trafficking, and M1 polarization of tumor-associated macrophages^17^. However, the immunomodulatory effects of anti-angiogenic agents in HCC have yet to be elucidated in a clinical setting.

In this study, we measured the concentrations of 16 soluble immune checkpoint proteins, using multiplexed fluorescent bead-based immunoassays, in plasma samples obtained from patients with advanced HCC. First, we performed multivariate analysis to determine whether levels of any soluble proteins were predictive of patient survival. We also conducted immunohistochemical (IHC) and immunofluorescence (IF) analysis to determine the localization of protiens of interest, both inside and at the margins of tumors. Lastly, we studied changes in the plasma levels of soluble proteins during the early stages of sorafenib treatment.

## Results

### Patient characteristics

The baseline characteristics of the 53 patients were described in Supplementary Table S1. In brief, the majority of patients were classified as Child-Pugh A (89%) and the remaining patients were classified as Child-Pugh B. Hepatitis C was the etiology in 66% of patients, hepatitis B in 13% of patients, and others causes, such as alcohol abuse, accounted for the remaining 21%. According to Barcelona Clinic Liver Cancer (BCLC) staging, advanced-stage HCC was present in 53% of the patients, with 17% of patients having microvascular metastases and 38% with distant metastases. Overall, 96% of patients had a history of other treatments; TACE was the most common treatment. According to the modified Response Evaluation Criteria in Solid Tumors (mRECIST), no patients showed a complete response to sorafenib treatment, 10 showed a partial response (PR), 10 had stable disease (SD), and 33 had progressive disease (PD).

### sBTLA levels at baseline were an independent factor predicting overall survival

**Figure 1:**
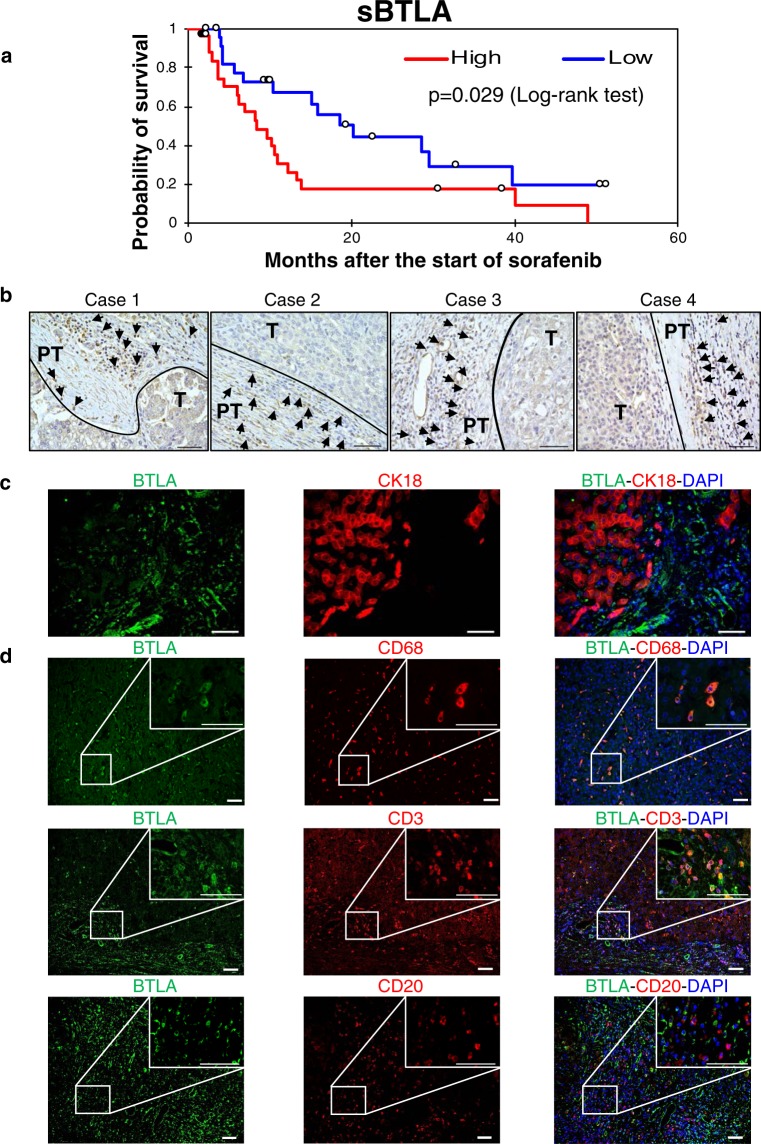
sBTLA levels predicts outcome of patients with HCC. (**a**) Kaplan-Meier survival analysis of 53 patients with advanced HCC at baseline including high levels of sBTLA and low levels of sBTLA. (**b**) Immunohistochemical staining of BTLA in liver tissues of four patients with HCC. Case 1 was a 21-year-old woman with HBV-related, huge but solitary HCC. Sorafenib was started 17 months after surgery, due to multiple intrahepatic recurrences and lung metastases. The plasma level of sBTLA was 807 pg/mL at the start of sorafenib treatment. Case 2 was a 73-year-old man with HCV-related, solitary HCC with a diameter of 45 mm. Sorafenib was started 12 months after surgery because of multiple intrahepatic recurrences and bone metastases. The plasma sBTLA level was 1,099 pg/mL prior to treatment. Case 3 was a 64-year-old man with alcoholic cirrhosis and solitary HCC at a diameter of 40 mm. Sorafenib was started 6 months after the operation, because of multiple intrahepatic recurrences and hilar lymph node metastasis. Plasma sBTLA levels were 311.08 pg/mL at the time of surgery and 98.9 pg/mL prior to treatment. Case 4 was a 77-year-old woman with HCV-related and solitary HCC with a diameter of 50 mm. Sorafenib treatment was initiated 5 months after surgery because of bone metastasis. PT: peri-tumoral, T: tumor. Scale bar, 50 μm. (**c**) Double immunofluorescent staining of BTLA (green) with CK18 (red), **(d)** CD68 (red), CD3 (red) or CD20 (red) in HCC tissues obtained from Case 3. Scale bar, 50 μm.

The concentrations of 16 soluble immune checkpoint proteins in plasma were measured for patients at baseline and at week 1, 2 and 4 after the start of sorafenib treatment (Supplementary Table S2). Univariate Cox regression analysis identified that hemoglobin <12.6 g/dL, serum albumin <3.5 g/dL, serum des-γ-carboxy prothrombin ≥200 mAU/mL and plasma sBTLA ≥395 pg/mL were significant factors associated with poor overall survival (OS) (Table 1). According to multivariate Cox regression analysis, only sBTLA ≥395 pg/mL was an independent risk factor associated with mortality with HR (95% CI) of 2.095 (1.040–4.220). In addition, the Kaplan-Meier survival curves for patients with high and low concentrations of sBTLA are shown in Fig. 1a. The median OS times were 8.4 months in the group of high sBTLA levels and 20.3 months in the group of low sBTLA levels (p = 0.029, log-rank test).

**Table 1:** Univariate and multivariate Cox regression analysis of factors associated with overall survival of patients with HCC.

Variables	Univariate analysis			Multivariate analysis		
	HR	95% CI	p value	HR	95% CI	p value
Age (≥71 years)	0.816	0.415–1.603	0.555			
Sex (male)	1.005	0.498–2.030	0.988			
HBV (positive)	1.376	0.525–3.611	0.516			
HCV (positive)	0.517	0.263–1.014	0.055			
Child-Pugh (≥6)	1.632	0.802–3.322	0.177			
Platelets (≥111 × 10^3^/mm3)	1.511	0.769–2.969	0.231			
Hemoglobin (≥12.6 g/dL)	0.490	0.000–0.971	0.041	0.630	0.315–1.260	0.192
Albumin (≥3.5 g/dL)	0.462	0.000–0.920	0.028	0.554	0.264–1.165	0.119
AST (≥55 U/L)	1.198	0.605–2.372	0.603			
ALT (≥40 U/L)	0.861	0.436–1.699	0.666			
AFP (≥200 ng/mL)	1.416	0.715–2.805	0.318			
AFP-L3% (≥5%)	1.766	0.890–3.505	0.104			
DCP (≥200 mAU/mL)	2.235	1.103–4.532	0.026	1.773	0.827–3.799	0.141
Sorafenib dose (800 mg/day)	0.945	0.474–1.884	0.873			
Vascular invasion or distant metastasis (positive)	1.533	0.776–3.029	0.219			
sBTLA (≥395 pg/mL)	2.105	1.062–4.170	0.033	2.095	1.040–4.220	0.038
sCD27 (≥4500 pg/mL)	0.751	0.383–1.471	0.404			
sCD28 (≥4000 pg/mL)	0.904	0.453–1.804	0.775			
sTIM-3 (≥5500 pg/mL)	0.862	0.442–1.682	0.663			
sHVEM (≥2500 pg/mL)	1.066	0.546–2.080	0.852			
sCD40 (≥650 pg/mL)	1.447	0.744–2.817	0.277			
sGITR (≥50 pg/mL)	1.246	0.640–2.427	0.517			
sLAG-3 (≥21500 pg/mL)	0.816	0.418–1.594	0.552			
sTLR-2 (≥900 pg/mL)	0.808	0.411–1.587	0.536			
sGITRL (≥180 pg/mL)	0.789	0.402–1.549	0.492			
sPD-1 (≥610 pg/mL)	1.521	0.764–3.029	0.232			
sCTLA-4 (≥30.5 pg/mL)	0.777	0.394–1.535	0.468			
sCD80 (≥82 pg/mL)	0.913	0.462–1.806	0.794			
sCD86 (≥825 pg/mL)	0.633	0.322–1.242	0.183			
sPD-L1 (≥42 pg/mL)	0.807	0.411–1.581	0.531			
sICOS (≥240 pg/mL)	0.894	0.457–1.749	0.744			

AFP, α-fetoprotein; AST, aspartate aminotransferase; ALT, alanine aminotransferase; DCP, des-γ-carboxy thrombin; HBV, hepatitis B virus; HCV, hepatitis C virus.

To further elucidate the localization of BTLA, 4 surgically resected sections (Fig. 1b) and 4 biopsy samples (Supplementary Fig. S1) were stained using antibodies against BTLA and other cell markers. We observed that BTLA was highly expressed in the peritumoral areas. BTLA was co-localized with a macrophage marker (CD68) and a T cell marker (CD3) but not with a hepatocyte marker (CK18) (Fig. 1c) and a B cell marker (CD20) (Fig. 1d).

### Changes of soluble immune checkpoint proteins during sorafenib treatment

At 1 week after commencing sorafenib treatment, the levels of sCD40 and sCD80 were significantly decreased compared with baseline values (p values were 0.006 and 0.045, respectively); no significant changes were observed in the other 14 proteins tested (Supplementary Fig. S2).

**Figure 2:**
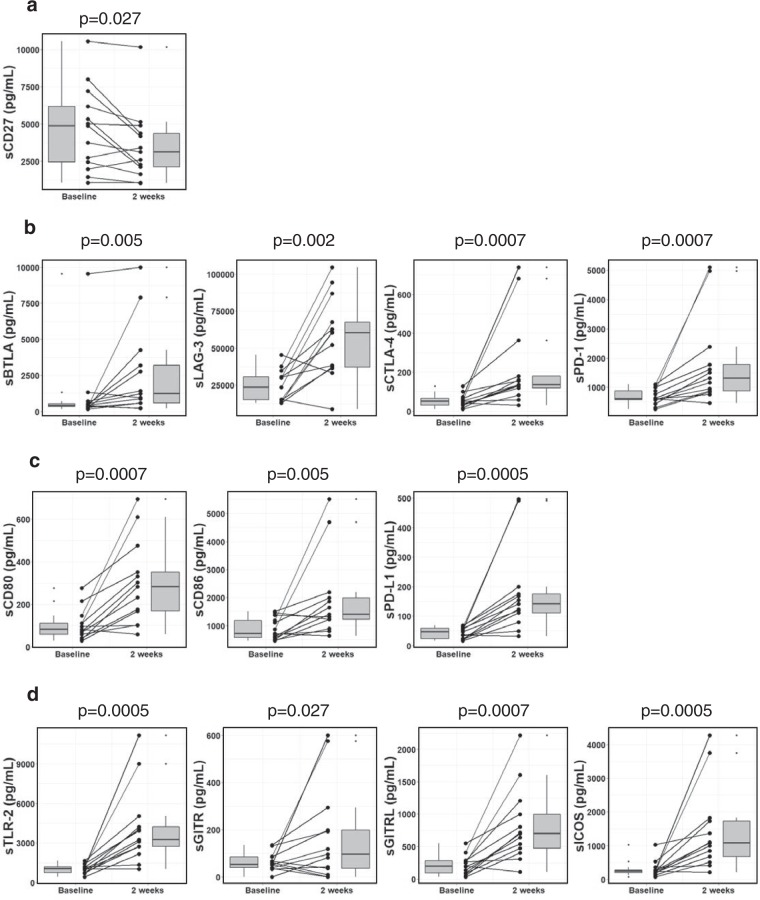
Profiling of soluble immune checkpoint protein levels after sorafenib treatment. Box plots of soluble proteins levels in patients with HCC at baseline and 2 weeks of treatment **(a)** sCD27; **(b)** sBTLA, sLAG-3, sCTLA-4 and sPD-1; **(c)** sCD80, sCD86 and sPD-L1; **(d)** sTLR, sGITR, sGITRL and sICOS. Ggplot2^47^ package was used for creating graphics. The vertical length of the box shows the interquartile range. The lines in the boxes shows the median values. The error bars show the minimum and maximum values (range). Wilcoxon signed-rank test was used. A p value of <0.05 was considered statistically significant.

After 2 weeks of treatment, the levels of numerous soluble checkpoint proteins had changed remarkably in comparison with baseline. Levels of sCD27, a member of the TNF receptor family that functions in activation and differentiation of T cells and boosting B cells^18^, were decreased by 0.64-fold with p = 0.027 (Fig. 2a). Conversely, levels of the soluble form of inhibitory receptors belonging to the immunoglobulin family were significantly increased, including sBTLA (2.95-fold, p = 0.005), sLAG-3 (2.56-fold, p = 0.002), sCTLA-4 (2.64-fold, p = 0.0007) and sPD-1 (2.12-fold, p = 0.0007) (Fig. 2b). In addition, levels of soluble forms of CTLA-4 and PD-1 ligands were increased, including sCD80 (3.43-fold, p = 0.0007), sCD86 (1.94-fold, p = 0.005) and sPD-L1 (3.05-fold, p = 0.0005) (Fig. 2c). TLR-2, GITR, GITRL and ICOS are known as stimulatory membrane-bound receptors, but previous studies have shown that their soluble forms may have inhibitory function^19–21^. In our study, the concentrations of sTLR-2, GITR, sGITRL and sICOS were significantly increased by 3.04-fold (p = 0.0005), 1.76-fold (p = 0.027), 3.58-fold (p = 0.0007) and 4.44-fold (p = 0.0005), respectively (Fig. 2d). The levels of sTIM-3, sHVEM, sCD40 and sCD28 were not significantly changed (Supplementary Fig. S3). At week 4 of treatment, we observed similar changes in the soluble forms of immune checkpoint proteins as at week 2 (Supplementary Fig. S4).

**Figure 3:**
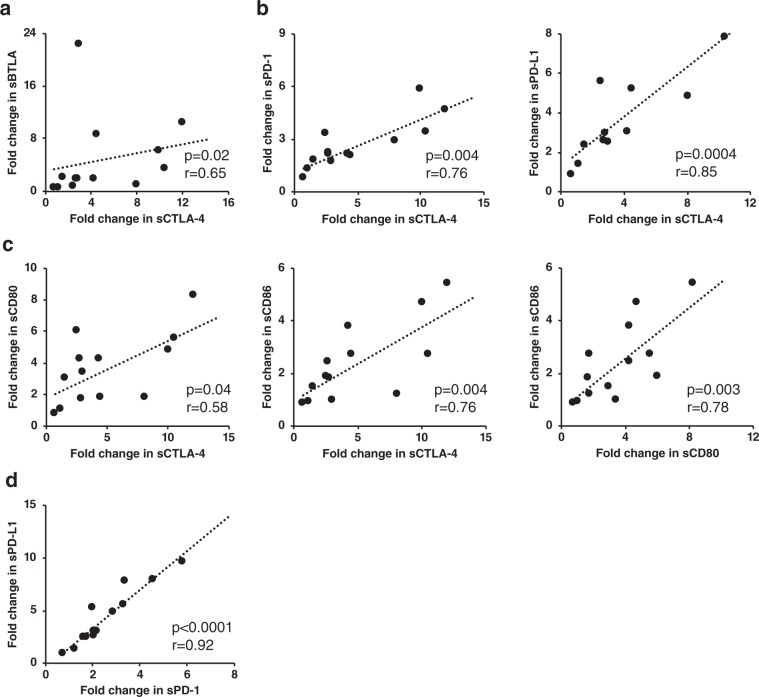
Relationship between fold-changes in soluble immune checkpoint protein levels at 2 weeks of treatment. Significant positive correlation between fold-change in (**a**) sCTLA-4 and sBTLA; (**b**) sCTLA-4 and sPD-1, and sCTLA-4 and sPD-L1; (**c**) sCTLA-4, sCD80, and sCD86 with each other; (**d**) sPD-1 and sPD-L1. Spearman’s rank correlation test was used. A p value of <0.05 was considered statistically significant.

The correlations between the fold-changes in soluble forms of immune checkpoint proteins at week 2 of treatment are shown in Fig. 3. The fold-change of sCTLA-4 was positively correlated with that of sBTLA (p = 0.02, r = 0.65) (Fig. 3a). Additionally, there were the positive correlations between the fold-change of sCTLA-4 and the fold-changes of sPD-1 (p = 0.004, r = 0.76) and sPD-L1 (p = 0.0004, r = 0.85) (Fig. 3b). The fold-change of sCTLA-4 was positively correlated with that of its soluble ligands sCD80 (p = 0.04, r = 0.58) and sCD86 (p = 0.004, r = 0.76); furthermore, the fold-changes of sCD80 and sCD86 were positively correlated with each other (p = 0.003, r = 0.78) (Fig. 3c). A positive correlation between the fold-changes in sPD-1 and sPD-L1 levels (p < 0.0001, r = 0.92) was also observed (Fig. 3d). Although the concentrations of soluble immune checkpoint proteins were not significantly changed at 1 week after start of sorafenib treatment, the correlation of their fold-changes were in the same pattern to the fold-changes at week 2 of treatment (Supplementary Fig. S5).

According to the response to sorafenib treatment, the patients were divided into the PD and SD + PR groups. No significant difference was observed in the fold-changes of 16 immune checkpoint protein levels between both groups at week 2 of treatment (Supplementary Fig. S6).

## Discussion

This study, for the first time, attempted simultaneous quantification of 16 soluble immune checkpoint proteins in patients with HCC during the early phase of treatment with sorafenib. These proteins included soluble forms of both stimulatory and inhibitory factors that regulate the activation and proliferation of T cells involved in the cancer-immunity cycle^22^. Using this extensive profiling, we identified significant changes not only for commonly studied inhibitory factors such as sPD-1, sPD-L1 or sCTLA-4^23–26^, but also for less well-studied checkpoint proteins.

First, we found that the plasma levels of sBTLA are an independent prognostic factor for the outcome of patients with advanced HCC (Table 1). Among significant factors associated with poor patient outcomes in our univariate analysis, low serum albumin concentrations represented impaired hepatic reserve, and high serum des-γ-carboxy prothrombin represented the invasive nature of the disease^27^. Our results suggest that the host’s anti-cancer immune response, at least partly represented by sBTLA levels, is also an important factor for patients’ survival. Although the number of patients was small and the p value was only slightly <0.05, the OS of patients with low levels of sBTLA was two times longer than that of patients with high levels of sBTLA (Fig. 1a). A previous study on pancreatic adenocarcinoma also indicated a negative correlation between plasma levels of sBTLA and OS^28^. BTLA is expressed in immune cells, including T cells^29^, and inhibits T cell activation in the late phase of immune responses^30^. In our study, BTLA was expressed in T cells, but also highly expressed in macrophages, which may possibly contributed to a weakened immune response by down-regulating macrophage activation^31^ (Fig. 1d).

Currently, numerous studies have reported that sPD-1, sPD-L1 and sCTLA-4 might play important roles in the initiation, promotion and progression of HCC^23–26^. However, the functions of these proteins are still unclear; soluble forms of inhibitory factors are not necessarily involved in negative immune regulation, and vice versa. For example, sPD-1 can compete against PD-1 by binding with PD-L1 and block PD-1/PD-L1 interactions to enhance anti-tumor responses^32^. In contrast, an inhibition of T cell proliferation was observed when co-culturing dendritic cells and T cells with sPD-1^33^. In this study, at week 1 of treatment, the levels of most soluble immune checkpoint proteins, including sCTLA-4 and sPD-1, showed no significant changes; only sCD40 and sCD80, which play key roles in the T cell–B cell interaction and T cell–antigen-presenting cell interaction, were significantly down-regulated (Supplementary Fig. S2). Nevertheless, at week 2 of treatment, significant increases were observed (Fig. 2b). Increases in levels of their ligands, including sCD80, sCD86 and sPD-L1, were also found (Fig. 2c). The soluble forms sCD80, sCD86 and sPD-L1 may inhibit T cell proliferation^34,35^ and can suppress induction of T cell apoptosis^36^. We speculate that these changes reflect the immunomodulatory effects of sorafenib on the tumor microenvironment.

We also investigated the levels of other soluble immune checkpoint proteins including the stimulatory factor CD27 and inhibitory factors such as BTLA and LAG-3. The soluble forms of CD27 can contribute to the activation of T cells and B cells^37,38^. In addition, previous studies have shown an increase in sBTLA levels in the blood of critically ill humans and mice^39^, and malignant melanoma cells express MHC-II, blinded sLAG-3 to up-regulate anti-apoptotic pathways^40^. Notably, at week 2 of sorafenib treatment, sCD27 levels were decreased from baseline (Fig. 2a). Conversely, the levels of soluble inhibitory factors BTLA and LAG-3 were increased (Fig. 2b). Although soluble inhibitory factors do not always have negative immune effects, we speculate that the immune systems may be exhausted at week 2 of sorafenib treatment. Interestingly, we found a correlation between elevated levels of sCTLA-4 with levels of sBTLA and sPD-1 (Fig. 3a,b). The mechanisms regulating these proteins remain unknown; however, these correlations suggest that similar mechanisms up-regulate expression of the soluble forms of CTLA-4, PD-1 and BTLA.

Among other soluble stimulatory factors, sTLR-2, GITR, sGITRL and sICOS levels were enhanced at week 2 of sorafenib treatment (Fig. 2d). Importantly, these proteins may down-regulate immune activation via distinct mechanisms. sTLR-2 regulates TLR-2 mediated inflammatory responses by disrupted the interaction of TLR-2 with its co-receptor^19^, while sGITRL derived from tumors may impair nature killer cell cytotoxicity and IFN-γ production^20^. Additionally, aberrant increases of sICOS levels with hepatitis C virus infection may reflect the dysregulation of T cell activation^21^. These results, together with the above results, suggest that the immune system is down-regulated, as revealed by the decrease of stimulatory factors and the increase of inhibitory factors.

Some combinations of anti-angiogenic agents and immune checkpoint inhibitors are currently under investigation. It is clinically important which anti-angiogenic agents are optimal partners for specific immune checkpoint inhibitors, and how much the optimal doses are when used in combination. The immunomodulatory effects of sorafenib may vary depending on the dosage. *In vivo* treatment with high-dose sorafenib may have negative effects on the immune microenvironment, such as an increase in PD-L1 expression^41^, or recruitment of myeloid-derived suppressor cells^42^, regulatory T cells^43^ and tumor-associated macrophages^44^. Additionally, in the present study, the increases in soluble forms of inhibitory factors observed at weeks 2 and 4 of treatment were less apparent in patients who received a reduced sorafenib dose compared with those in patients who did not receive a reduced dose (Supplementary Fig. S7). However, the relationship of sorafenib dose intensity and plasma parameters was indeterminate.

The levels of some soluble immune checkpoint proteins were elevated immediately after sorafenib administration; however, the fluctuation of these proteins in the PD and SD + PR groups showed no difference (Supplementary Fig. S6). These data suggested that the prompt change in the levels of soluble immune checkpoints is a direct immune reaction rather than an indirect reaction mediated by tumor necrosis caused by sorafenib administration.

This study has some limitations. First, it is a single-arm design of a real-world, retrospective study. Also, plasma samples were not stored for all consecutive sorafenib-treated patients with advanced HCC. However, we observed similar changes in levels of soluble immune checkpoint proteins at week 2 and 4 of treatment. Second, although we observed marked changes in the concentrations of the 16 proteins, we could not identify a specific pattern that correlated with treatment outcomes or other clinical factors, due to the small number of patients. Third, plasma samples were not necessarily collected at the exact time-points of surgery or biopsy, and the sorafenib treatment was occasionally initiated a long time after surgery; therefore, it was difficult to determine the correlation of the soluble and membrane-bound forms of immune checkpoint proteins.

In conclusion, our data revealed that levels of sBTLA can be used as a potential marker to predict OS in patients with advanced HCC. Further, patients with high levels of sBTLA at baseline had shorter survival times than those with low levels. After 2 weeks of sorafenib treatment, the increased levels of inhibitory factors, including sPD-1, sCTLA-4, sBTLA, and sLAG-3 and the decreased levels of stimulatory factors, such as sCD27, possibly reflect the dynamic changes of the immune systems in patients with HCC. Further investigation is necessary in a large group of patients, in patients with different etiologies or in patients at earlier stages to explore potential biomarkers that correlate with HCC initiation, promotion and progression.

## Patients and Methods

### Patients

Between September 2009 and December 2017, we initiated sorafenib treatment in 110 patients with advanced HCC at our institute. In this study, 53 patients who had stored baseline plasma samples collected at the start of treatment [36 males and 17 females; median age, 71 (range, 21–89) years] were enrolled. Patients were diagnosed with HCC by radiology and/or biopsy following the guidelines^5,6^. The stage of HCC was determined by the BCLC system. The patients started sorafenib (Nexavar, Bayer Yakuhin, Ltd., Osaka, Japan) at an oral dose of 400 mg, twice daily. The dose was reduced in 25 patients within the first week of treatment according to the manufacturer’s recommendations because of adverse reactions to dermatological, hematological or gastrointestinal toxicities. All patients supplied informed consent, and the study was conducted in accordance with the Helsinki Declaration and approved by the Ethical Committee of Osaka City University.

### Soluble immune checkpoint protein assays

Plasma samples were collected at baseline (within 28 days prior to initiating sorafenib treatment, n = 53), at week 1 (n = 32), at week 2 (n = 13) and at week 4 (n = 12) of sorafenib treatment. The levels of 16 soluble immune checkpoint proteins were measured using multiplexed fluorescent bead-based immunoassays with the Milliplex Map Kit (EMD Millipore Corporation, Massachusetts, USA) and Bio-Rad’s Luminex Bio-Plex- 200 system. These proteins include: soluble (s) B- and T-lymphocyte attenuator (sBTLA), sCD27, sCD28, soluble T-cell immunoglobulin and mucin domain-3 (sTIM-3), soluble herpes virus entry mediator (sHVEM), sCD40, soluble glucocorticoid-induced TNFR-related (sGITR), soluble lymphocyte-activation gene 3 (sLAG-3), soluble toll-like receptor 2 (sTLR-2), soluble glucocorticoid-induced TNFR-related ligand (sGITRL), soluble programmed cell death protein 1 (sPD-1), soluble cytotoxic T-lymphocyte associated antigen 4 (sCTLA-4), sCD80, sCD86, soluble programmed cell death-ligand 1 (sPD-L1) and soluble inducible T-cell co-stimulator (sICOS). According to the manufacturer’s instructions, 12.5 μL of plasma were used for each measurement and all samples were assayed in duplicate; mean values were used for further analysis. For values lower than the limit of detection, we used 10% of the lowest recorded values as a substitute. We investigated the association between OS and clinical parameters, including the baseline levels of these soluble checkpoint proteins.

### Immunohistochemical and immunofluorescence analysis

Histological evaluations were performed for four patients whose surgical specimens were obtained and four patients whose biopsy specimens were stored. Intra- and peri-tumoral sections were assessed by IHC and IF, as previously described^45^, using the following primary antibodies and dilution ratios: anti-BTLA (Abcam, rabbit polyclonal anti-CD272 antibody, ab181406, IHC 1:500, IF 1:60), anti-CK18 (Novous, NB500–353, 1:100), anti-CD3 (Dako, Clone F7.2.38, 1:50), anti-CD20 (Dako, Clone L26, 1:200) and anti-CD68 (Dako, clone PG-M1, 1:80). The tissue sections (5 μm-thick) from formalin-fixed, paraffin-embedded blocks were deparaffinized in xylene and dehydrated in decreasing concentrations of ethanol. Antigen retrieval was performed by autoclaving for 15 minutes at 120 °C in 0.01 M citrate buffer, pH 6.0. The sections were incubated overnight with primary antibodies at 4 °C. For endogenous peroxidases, the samples were incubated with 3% hydrogen peroxidase in absolute methanol for 15 minutes. The secondary antibodies used were Envision system-HRP labelled polymer anti-rabbit (Dako, Agilent Technologies, California, USA), Alexa Fluor 594 goat anti-mouse, and Alexa Flour 488 goat anti-rabbit (Invitrogen, Thermo Fisher Scientific, Massachusetts, USA). 3,3′-Diaminobenzindine (Dako), a chromagen applied to visualize antibody/antigen complexes, was used for immunohistochemical staining. The stained sections were analyzed with a BZ-X710 microscope (Kyence, Osaka, Japan).

### Statistical analysis

Analysis was conducted in R^46^ and figures were produced using the package ggplot2^47^. Cox proportional hazards models were used to analyze factors associated with OS in patients with advanced HCC. Variables exhibiting significant differences in univariate Cox regression analysis were subjected to stepwise multivariate Cox regression analysis. Kaplan-Meier analysis and log-rank tests were used to estimate and compare OS between two groups. Wilcoxon signed-rank tests were used to compare changes in the soluble immune checkpoint concentrations during the early treatment period. Correlations of fold-changes in levels of two proteins were determined using Spearman’s rank correlation test. A p value of <0.05 was considered statistically significant.

### Ethics approval

All patients supplied informed consent, and the study was conducted in accordance with the Helsinki Declaration and approved by the Ethical Committee of Osaka City University.

### Consent for publication

No consent was involved in this publication.

## Supplementary information

Supplementary information.

## Data Availability

All data and materials generated during and/or analyzed during the current study are available from the corresponding author on reasonable request.

## References

[1] Sharma P, Allison JP (2015). The future of immune checkpoint therapy. Sci..

[2] Buder-Bakhaya K, Hassel JC (2018). Biomarkers for Clinical Benefit of Immune Checkpoint Inhibitor Treatment-A Review From the Melanoma Perspective and Beyond. Front. Immunol..

[3] Gu D, Ao X, Yang Y, Chen Z, Xu X (2018). Soluble immune checkpoints in cancer: production, function and biological significance. J. Immunother. Cancer..

[4] Torre LA (2015). Global cancer statistics, 2012. CA Cancer J. Clin..

[5] Marrero JA (2018). Diagnosis, Staging, and Management of Hepatocellular Carcinoma: 2018 Practice Guidance by the American Association for the Study of Liver Diseases. Hepatology..

[6] European Association for the Study of the Liver. (2018). EASL Clinical Practice Guidelines: Management of hepatocellular carcinoma. J. Hepatol..

[7] Llovet JM (2008). Sorafenib in advanced hepatocellular carcinoma. N. Engl. J. Med..

[8] Kudo M (2018). Lenvatinib versus sorafenib in first-line treatment of patients with unresectable hepatocellular carcinoma: a randomised phase 3 non-inferiority trial. Lancet..

[9] Bruix J (2017). Regorafenib for patients with hepatocellular carcinoma who progressed on sorafenib treatment (RESORCE): a randomised, double-blind, placebo-controlled, phase 3 trial. Lancet..

[10] Abou-Alfa GK (2018). Cabozantinib in Patients with Advanced and Progressing Hepatocellular Carcinoma. N. Engl. J. Med..

[11] Zhu AX (2019). Ramucirumab after sorafenib in patients with advanced hepatocellular carcinoma and increased α-fetoprotein concentrations (REACH-2): a randomised, double-blind, placebo-controlled, phase 3 trial. Lancet Oncol..

[12] Yau T (2019). Nivolumab in Advanced Hepatocellular Carcinoma: Sorafenib-Experienced Asian Cohort Analysis. J. Hepatol..

[13] Zhu AX (2018). Pembrolizumab in patients with advanced hepatocellular carcinoma previously treated with sorafenib (KEYNOTE-224): a non-randomised, open-label phase 2 trial. Lancet Oncol..

[14] Makker V (2019). Lenvatinib plus pembrolizumab in patients with advanced endometrial cancer: an interim analysis of a multicentre, open-label, single-arm, phase 2 trial. Lancet Oncol..

[15] Cheng A-L (2019). Efficacy and safety results from a ph III study evaluating atezolizumab (atezo) + bevacizumab (bev) vs sorafenib (Sor) as first treatment (tx) for patietns (pts) with unresectable hepatocellular carcinoma (HCC). Ann. Oncol..

[16] Xu J-M (2018). Anti-programmed death-1 antibody SHR-1210 (S) combined with apatinib (A) for advanced hepatocellular carcinoma (HCC), gastric cancer (GC) or esophagogastric junction (EGJ) cancer refractory to standard therapy: A phase 1 trial. J. Clin. Oncology..

[17] Lin YY (2018). Immunomodulatory Effects of Current Targeted Therapies on Hepatocellular Carcinoma: Implication for the Future of Immunotherapy. Semin. Liver Dis..

[18] Denoeud J, Moser M (2011). Role of CD27/CD70 pathway of activation in immunity and tolerance. J. Leukoc. Biol..

[19] Henrick BM, Yao XD, Taha AY, German JB, Rosenthal KL (2016). Insights into Soluble Toll-Like Receptor 2 as a Downregulator of Virally Induced Inflammation. Front. Immunol..

[20] Baltz KM (2008). Neutralization of tumor-derived soluble glucocorticoid-induced TNFR-related protein ligand increases NK cell anti-tumor reactivity. Blood..

[21] Wang D (2013). Aberrant production of soluble inducible T-cell co-stimulator (sICOS) and soluble programmed cell death protein 1 (sPD-1) in patients with chronic hepatitis C. Mol. Med. Rep..

[22] Chen DS, Mellman I (2013). Oncology meets immunology: the cancer-immunity cycle. Immunity..

[23] Cheng HY (2014). Circulating programmed death-1 as a marker for sustained high hepatitis B viral load and risk of hepatocellular carcinoma. Plos One..

[24] Zeng Z (2011). Upregulation of circulating PD-L1/PD-1 is associated with poor post-cryoablation prognosis in patients with HBV-related hepatocellular carcinoma. Plos One..

[25] Chang B (2019). The correlation and prognostic value of serum levels of soluble programmed death protein 1 (sPD-1) and soluble programmed death-ligand 1 (sPD-L1) in patients with hepatocellular carcinoma. Cancer Immunol. Immunother..

[26] Liu Q (2017). Soluble cytotoxic T-lymphocyte antigen 4: a favorable predictor in malignant tumors after therapy. Onco Targets Ther..

[27] Inagaki Y (2011). Clinical and molecular insights into the hepatocellular carcinoma tumour marker des-γ-carboxyprothrombin. Liver Int..

[28] Bian B (2019). Prognostic significance of circulating PD-1, PD-L1, pan-BTN3As, BTN3A1 and BTLA in patients with pancreatic adenocarcinoma. Oncoimmunology..

[29] Sedy JR (2005). B and T lymphocyte attenuator regulates T cell activation through interaction with herpesvirus entry mediator. Nat. Immunol..

[30] Watanabe N (2003). BTLA is a lymphocyte inhibitory receptor with similarities to CTLA-4 and PD-1. Nat. Immunol..

[31] Yang C (2013). Expression of B and T lymphocyte attenuator (BTLA) in macrophages contributes to the fulminant hepatitis caused by murine hepatitis virus strain-3. Gut..

[32] He L (2005). Blockade of B7-H1 with sPD-1 improves immunity against murine hepatocarcinoma. Anticancer. Res..

[33] Kuipers H (2006). Contribution of the PD-1 ligands/PD-1 signaling pathway to dendritic cell-mediated CD4+ T cell activation. Eur. J. Immunol..

[34] Kakoulidou M, Giscombe R, Zhao X, Lefvert AK, Wang X (2007). Human Soluble CD80 is generated by alternative splicing, and recombinant soluble CD80 binds to CD28 and CD152 influencing T-cell activation. Scand. J. Immunol..

[35] Fló J, Tisminetzky S, Baralle F (2001). Codelivery of DNA coding for the soluble form of CD86 results in the down-regulation of the immune response to DNA vaccines. Cell Immunol..

[36] Frigola X (2012). Soluble B7-H1: differences in production between dendritic cells and T cells. Immunol. Lett..

[37] Dang LV (2012). Soluble CD27 induces IgG production through activation of antigen-primed B cells. J. Intern. Med..

[38] Huang J (2013). Soluble CD27-pool in humans may contribute to T cell activation and tumor immunity. J. Immunol..

[39] Monaghan SF (2018). Changes in the process of alternative RNA splicing results in soluble B and T lymphocyte attenuator with biological and clinical implications in critical illness. Mol. Med..

[40] Hemon P (2011). MHC class II engagement by its ligand LAG-3 (CD223) contributes to melanoma resistance to apoptosis. J. Immunol..

[41] Chen Y (2017). Overcoming sorafenib evasion in hepatocellular carcinoma using CXCR4-targeted nanoparticles to co-deliver MEK-inhibitors. Sci. Rep..

[42] Lam W (2015). PHY906(KD018), an adjuvant based on a 1800-year-old Chinese medicine, enhanced the anti-tumor activity of Sorafenib by changing the tumor microenvironment. Sci. Rep..

[43] Zhou SL (2016). Tumor-Associated Neutrophils Recruit Macrophages and T-Regulatory Cells to Promote Progression of Hepatocellular Carcinoma and Resistance to Sorafenib. Gastroenterology..

[44] Zhang W (2010). Depletion of tumor-associated macrophages enhances the effect of sorafenib in metastatic liver cancer models by antimetastatic and antiangiogenic effects. Clin. Cancer Res..

[45] Thuy, 1. T. *et al*. Promotion of liver and lung tumorigenesis in DEN-treated cytoglobin-deficient mice. *Am J Pathol*. **179**, 1050–1060 (2011). 10.1016/j.ajpath.2011.05.006 PMC3157247 21684245

[46] R Core Team. R: A language and environment for statistical computing. *R Foundation for Statistical Computing*. Vienna, Austria. http://www.R-project.org/ (2019)

[47] Wickham, H. ggplot2: elegant graphics for data analysis. *Springer-Verlag New York* (2016).

