# Supplementary material for: Clinical significance of circulating soluble immune checkpoint proteins in sorafenib-treated patients with advanced hepatocellular carcinoma
Source: Sci Rep. 2020 Feb 25;10:3392. doi: 10.1038/s41598-020-60440-5 (PMC7042216; doi:10.1038/s41598-020-60440-5)
Supplement: Supplementary file 1 — Supplementary information. [file 41598_2020_60440_MOESM1_ESM.pdf]

Supplementary materials

Title: Clinical significance of circulating soluble immune checkpoint proteins in sorafenib-treated patients with advanced hepatocellular carcinoma

Authors: Dong Minh Phuong[1], Masaru Enomoto[1], Le Thi Thanh Thuy[1], Hoang Hai[1], Vu Ngọc Hieu[1], Dinh Viet Hoang[1], Ayako Iida-Ueno[1], Naoshi Odagiri[1], Yuga Amano-Teranishi[2], Atsushi Hagihara[1], Hideki Fujii[1], Sawako Uchida-Kobayashi[1], Akihiro Tamori[1] and Norifumi Kawada[1]

[1] Department of Hepatology, Graduate School of Medicine, Osaka City University, Osaka 5458585, Japan

[2] Department of Hepatology, Osaka City General Hospital, Osaka 5340021, Japan

Table of Contents

Supplementary Table S1 ……………………………………………………......2

Supplementary Table S2 …………………………………………….………......3

Supplementary Figure S1……………………………………………………......4

Supplementary Figure S2 …………………………………………………….....5

Supplementary Figure S3……………………………………….………………6

Supplementary Figure S4 …………………………………………………….....7

Supplementary Figure S5 …………………………………….………...….8

Supplementary Figure S6 …………………………………….………...….9

Supplementary Figure S7 …………………………………….………...…10

Supplementary Table S1: Characteristics of patients at baseline

Characteristics	Value (n=53)*
Age (years)	71 (63–77)
Sex (male/female)	36/17
Etiology (HBV/HCV/others)	7/35/11
Child-Pugh (A/B)	47/6
BCLC stage (intermediate/advanced)	25/28
Previous HCC therapies (operation/ablation/TACE/TAI)	7/5/46/15
Response to sorafenib (PD/SD+PR)	33/20
Duration of treatment (days)	61 (28.5–149.5)
Overall survival (months)	10.9 (5.6–29.4)

*Results are shown as number of patients or median (interquartile range)

Supplementary Table S2: The concentrations* of soluble immune checkpoint proteins in advanced HCC patients following sorafenib treatment.

	Soluble proteins	Baseline (53 patients)	Week 1 (32 patients)	Week 2 (13 patients)	Week 4 (12 patients)
1	sBTLA	660.8 ± 1317.5	660.7 ± 816.0	2639.3 ± 3084.1	2528.0 ± 3588.4
2	sCD27	5128.1 ± 2803.2	4673.0 ± 2731.9	3546.3 ± 2419.0	2765.5 ± 2015.7
3	sCD28	4635.0 ± 2756.9	4569.7 ± 2213.2	4863.8 ± 2702.5	4226.1 ± 1649.6
4	sTIM-3	5723.5 ± 2199.7	5327.6 ± 2064.3	5147.6 ± 2169.3	4231.8 ± 2132.8
5	sHVEM	2675.4 ± 978.0	2516.5 ± 825.4	2481.0 ± 994.3	2217.1 ± 1248.4
6	sCD40	681.0 ± 228.0	608.1 ± 166.3	679.4 ± 232.7	622.6 ± 289.9
7	sGITR	78.4 ± 121.4	75.7 ± 86.8	173.3 ± 204.1	145.7 ± 124.0
8	sLAG-3	25103.3 ±16699.2	24671.2 ± 13345.7	57198.0 ± 27049.9	61624.6 ± 33434.5
9	sTLR-2	1134.6 ± 941.5	1226.6 ± 959.5	4140.7 ± 2899.2	4151.8 ± 3118.6
10	sGITRL	253.0 ± 255.1	244.4 ± 258.6	824.2 ± 573.4	800.6 ± 626.6
11	sPD-1	790.2 ± 591.0	787.9 ± 595.7	1827.5 ± 1513.6	1652.0 ± 1367.7
12	sCTLA-4	46.0 ± 38.7	45.9 ± 46.3	228.8 ± 228.4	190.7 ± 171.0
13	sCD80	106.7 ± 72.5	92.6 ± 66.6	299.1 ± 196.4	273.8 ± 187.7
14	sCD86	915.3 ± 476.8	939.8 ± 478.7	1953.3 ± 1481.0	1833.9 ± 1573.7
15	sPD-L1	79.9 ± 150.3	76.5 ± 113.8	180.2 ± 147.6	155.6 ± 126.9
16	sICOS	474.3 ± 767.0	534.6 ± 650.6	1445.9 ± 1241.6	1317.0 ± 1156.8

* pg/mL, mean ± SD

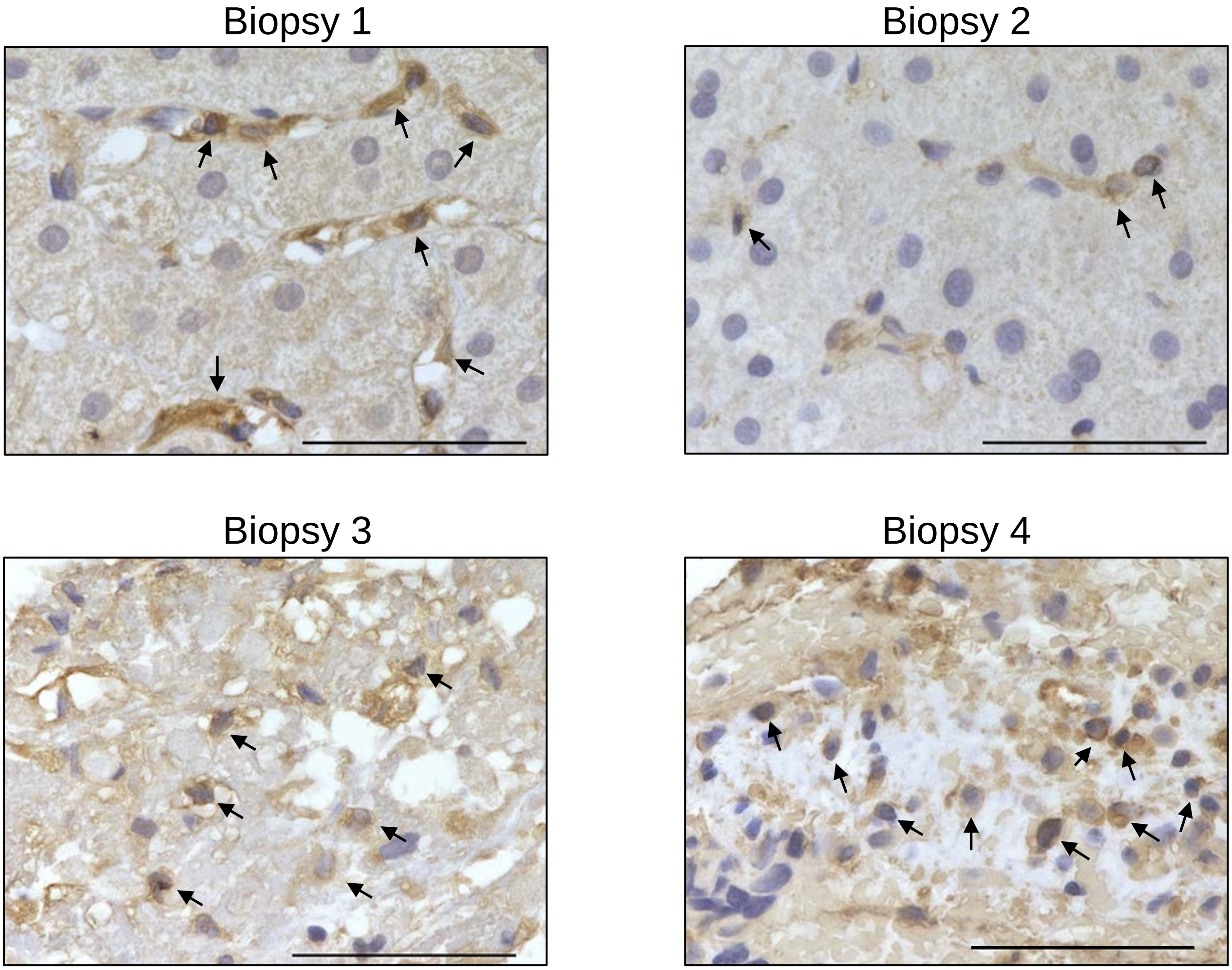

Supplementary Figure S1. Immunohistochemical staining for BTLA in biopsy sections of patients with HCC. Plasma levels of sBTLA prior to sorafenib treatment in biopsies 1 through 4 were 134.6, 41.34, 376.66, and 438.24 pg/mL. Scale bar, 50 μm.

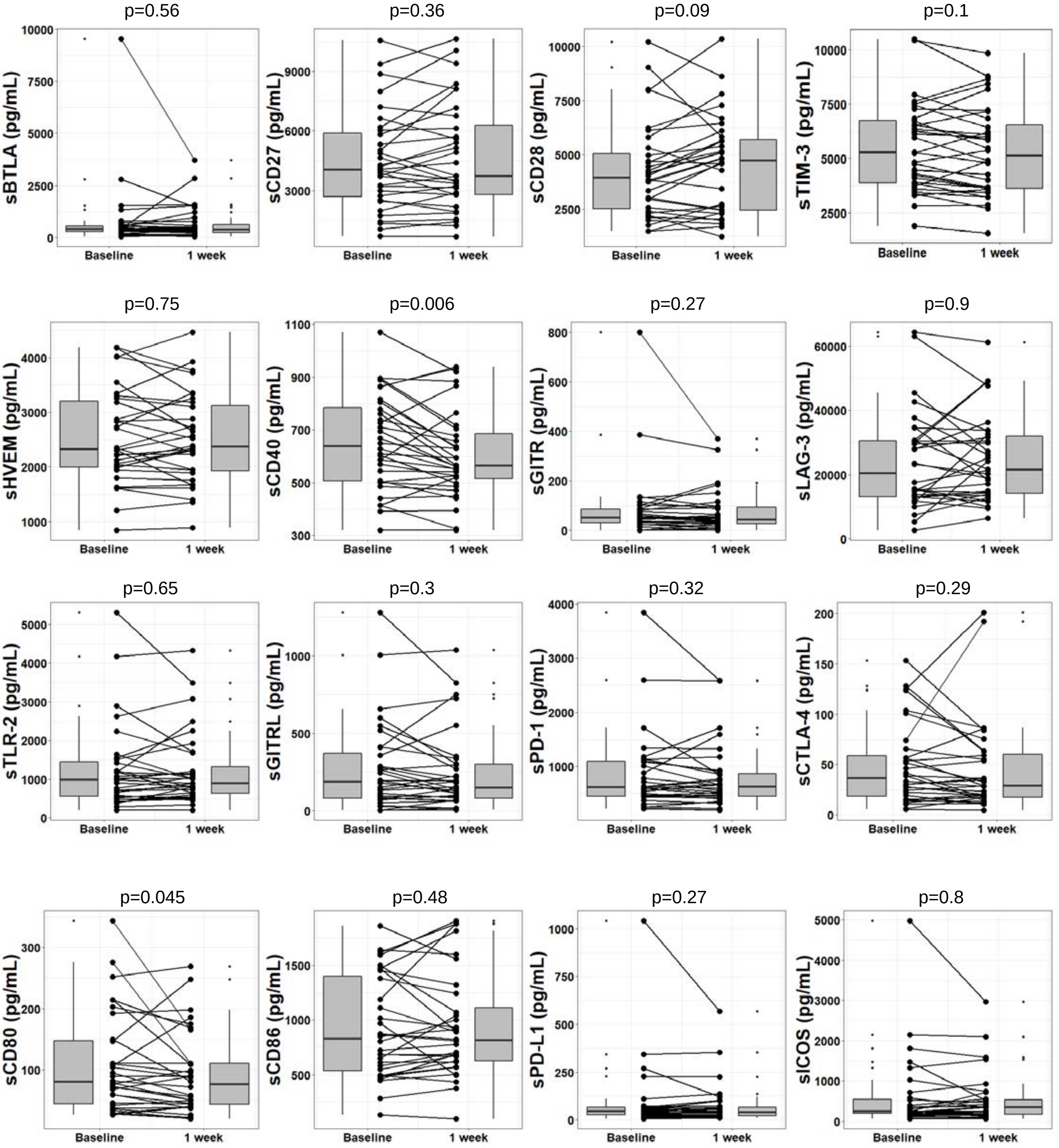

Supplementary Figure S2. Changes of soluble immune checkpoint protein levels after 1 week of sorafenib treatment. Box plots display sBTLA, sCD27, sCD28, sTIM-3, sHVEM, sCD40, sGITR, sLAG-3, sTLR-2, sGITRL, sPD-1, sCTLA-4, sCD80, sCD86, sPD-L1, and sICOS levels in HCC patients at baseline and week 1 of treatment. Ggplot2[47] package was used for creating graphics. The vertical lengths of the boxes show the interquartile ranges and the lines in the boxes show median values. The error bars show the minimum and maximum values (range). Wilcoxon signed-rank test were used. A p value of <0.05 was considered statistically significant.

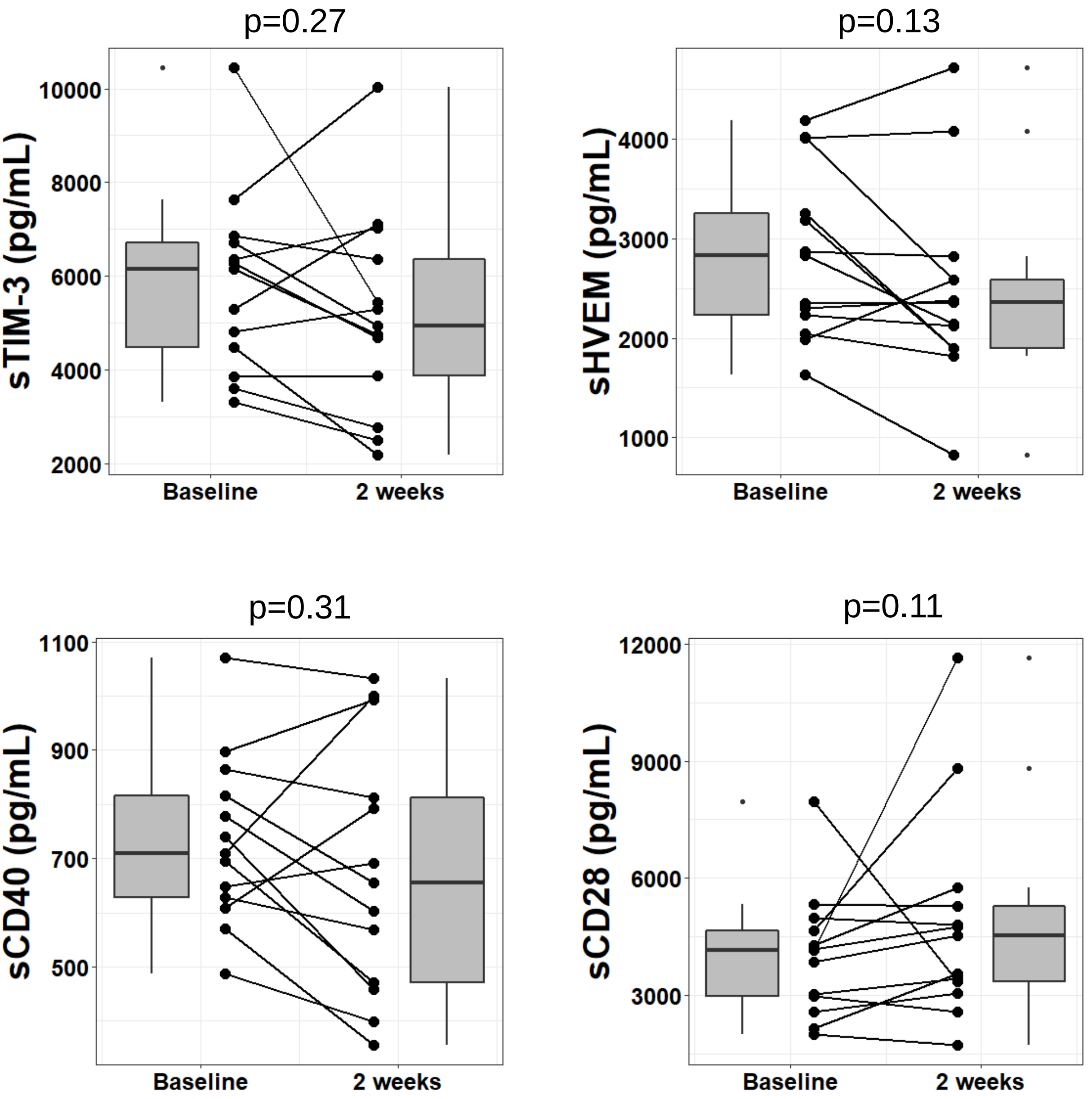

Supplementary Figure S3. Changes of soluble immune checkpoint protein levels after 2 weeks of sorafenib treatment. Box plots display sTIM-3, sHVEM, sCD40, and sCD28 levels in HCC patients at baseline and at week 2 of treatment. Ggplot2[47] package was used for creating graphics. The vertical lengths of the boxes show interquartile ranges and the lines in the boxes shows median values. The error bars show the minimum and maximum values (range). Wilcoxon signed-rank test were used. A p value of <0.05 was considered statistically significant.

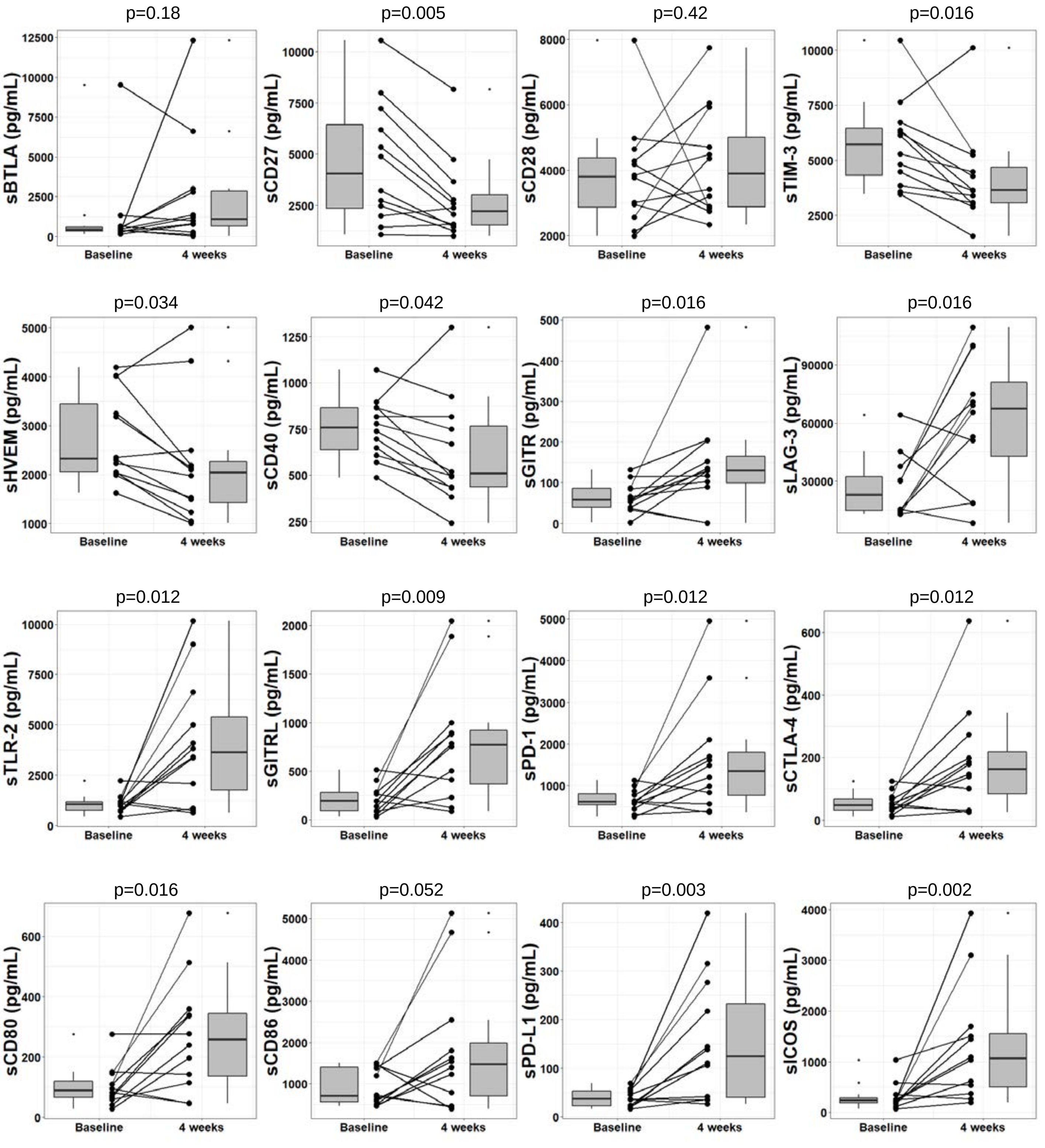

Supplementary Figure S4. Changes of soluble immune checkpoint protein levels after 4 weeks of sorafenib treatment. Box plots display sBTLA, sCD27, sCD28, sTIM-3, sHVEM, sCD40, sGITR, sLAG-3, sTLR-2, sGITRL, sPD-1, sCTLA-4, sCD80, sCD86, sPD-L1, and sICOS levels in HCC patients at baseline and at week 4 of treatment. Ggplot2[47] package was used for creating graphics. The vertical lengths of the boxes show the interquartile ranges and the lines in the boxes shows median values. The error bars show the minimum and maximum values (range). Wilcoxon signed-rank test were used. A p value of <0.05 was considered statistically significant.

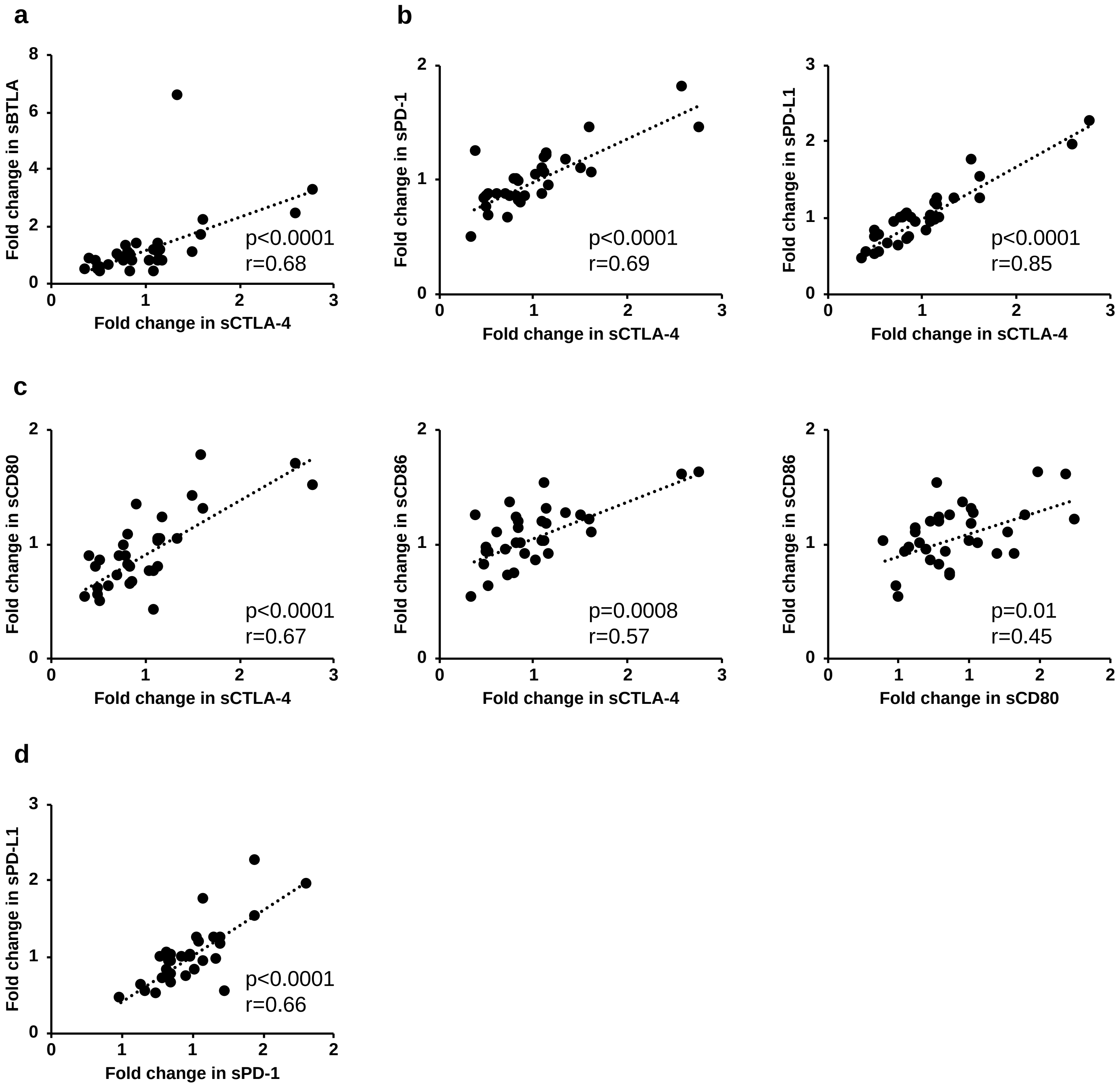

Supplementary Figure S5. Relationship between fold-changes in soluble immune checkpoint protein levels after 1 week of treatment. Significant positive correlations between fold-changes in **(a)** sCTLA-4 and sBTLA; **(b)** sCTLA-4 and sPD-1, and sCTLA-4 and sPD-L1; **(c)** sCTLA-4, sCD80, and sCD86 with each other; **(d)** sPD-1 and sPD-L1. Spearman's rank correlation test were used. A p value of <0.05 was considered statistically significant.

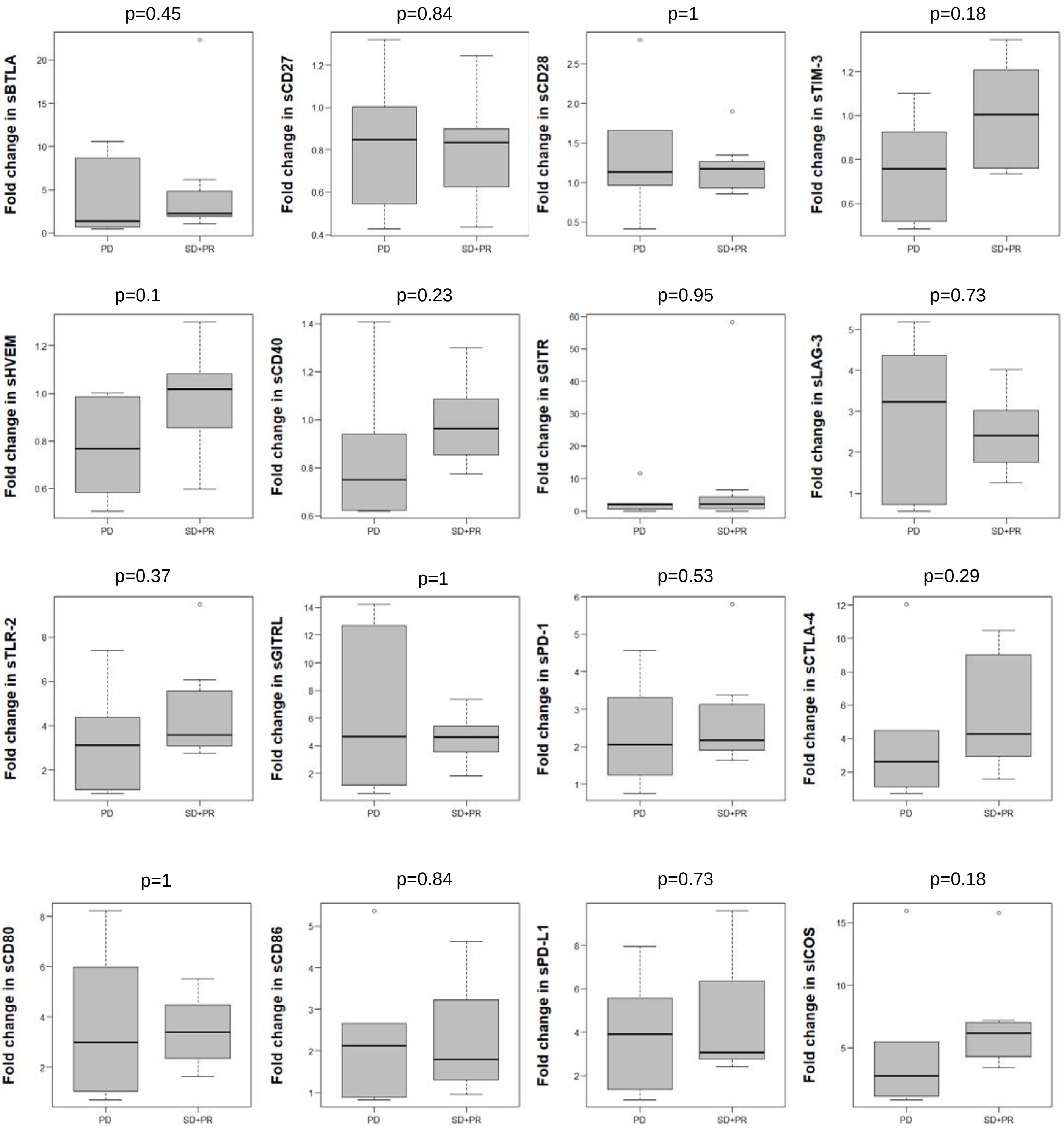

Supplementary Figure S6. Changes in soluble immune checkpoint protein levels following sorafenib treatment according to response. Boxplots display the fold-changes in the levels of sBTLA, sCD27, sCD28, sTIM-3, sHVEM, sCD40, sGITR, sLAG-3, sTLR-2, sGITRL, sPD-1, sCTLA-4, sCD80, sCD86, sPD-L1, and sICOS in patients with PD (progressive disease) or SD + PR (stable disease + partial response) at week 2 compared with baseline. The vertical lengths of the boxes show the interquartile ranges and the lines in the boxes show the median values. The error bars show the minimum and maximum values (range). The Mann–Whitney U test was used. A p value of <0.05 was considered statistically significant.

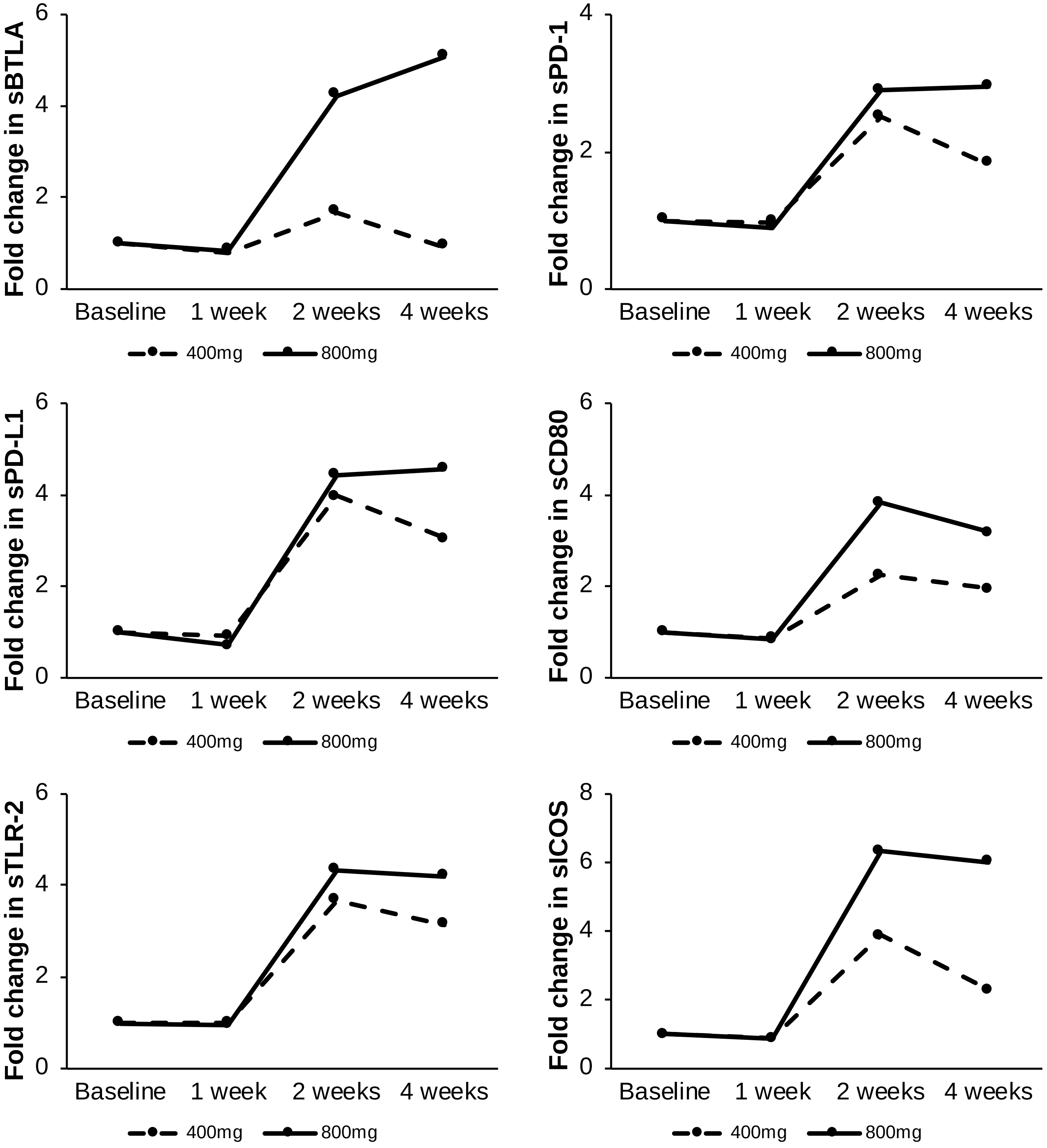

Supplementary Figure S7. Dose dependently changes of soluble immune checkpoint protein levels following sorafenib treatment. Graphs showed the fold changes of sBTLA, sPD-1, sPD-L1, sCD80, sTLR-2 and sICOS levels in patients who received an initial dose of sorafenib (800 mg) and in patients with immediate dose reduction (to 400 mg) at baseline and at week 1, 2, and 4 of treatment.
